# Prevalence of frailty and associated factors in older adults seeking care at Swedish emergency departments

**DOI:** 10.1186/s12877-023-04545-2

**Published:** 2023-12-04

**Authors:** Ann-Sofie Källberg, Lena M Berg, Sara Skogli, Charlotte Bjurbo, Åsa Muntlin, Anna Ehrenberg

**Affiliations:** 1 School of Health and Welfare, Dalarna University, Falun, SE-791 88 Sweden; 2 Department of Emergency Medicine, Falun Hospital and Centre for Clinical Research Dalarna, Nissers väg 3, Falun, SE-791 82 Sweden; 3 Department of Internal Medicine, Falun Hospital, Falun, SE-791 82 Sweden; 4 Department of Emergency Care and Internal Medicine, Uppsala University Hospital, entrance 40, level 5, Uppsala, SE-751 85 Sweden; 5 Department of Medical Sciences, Uppsala University, Uppsala University Hospital, entrance 40, level 5, Uppsala, SE-751 85 Sweden

**Keywords:** Emergency service hospital, Frailty, Older adult, Screening

## Abstract

**Background:**

Internationally, prolonged length of stay for older adults in the emergency department (ED) is associated with increased risk of in-hospital adverse events. In Sweden patients 65 years and older account for 35% of emergency visits, and according to consensus from an international expert group, all persons over 70 should be screened for frailty. This is not routinely done in Swedish EDs, and therefore, knowledge about prevalence, characteristics and clinical outcomes associated with frailty is limited.

**Aim:**

To describe the prevalence of frailty and associated factors in older adults seeking care at Swedish EDs.

**Methods:**

The study has a cross-sectional design. Data was collected at three hospital-based EDs, varying in level and size of setting, for one month. Patients age 70 and older presenting at the EDs and agreed to participate were screened for frailty using the FRail Elderly Support researcH group (FRESH) instrument. Data were analysed using descriptive statistics to assess the distribution of patient characteristics and clinical outcomes. Multivariate logistic regression was used to model the association between frailty and demographic characteristics, and Cox regression was used to model the association between frailty and clinical outcomes.

**Results:**

A total of 3101 patients were eligible for inclusion; of these, 984 (32%) were included and screened for frailty. Of the final sample, 57.3% were assessed as frail. Characteristics significantly associated with frailty were living in a residential care facility, age (> 80 years), being a woman and arriving with emergency medical service (EMS). There was a significant association between frailty and admittance to in-hospital care.

**Conclusion:**

Our study shows a high prevalence of frailty in older people. Factors associated with frailty were living in a residential care facility, age ≥ 80 years, being a woman and arriving with EMS to the ED and being admitted to in-hospital care. Frailty screening should be incorporated in the triage system to identify frail patients who need tailored interventions. More studies using the FRESH instrument are needed to further confirm our findings and to develop the methods for screening for frailty in the ED.

## Background

People in many countries are expected to live longer [1, 2] the number of persons aged 80 years or older is expected to triple during the next 30 years [1]. Many older adults are living with multimorbidity and frailty, and consequently, the need for emergency care is increasing and will likely increase even more in the future [3–5]. Internationally, older adults account for more emergency department (ED) visits than other age groups [3, 6]. In Sweden, patients 65 years and older account for 35% of the ED visits, and patients over the age of 80 years have a prolonged length of stay (LOS) compared with other patients [7].

However, today`s highly specialized emergency care is poorly adapted to the comprehensive care needs of frail older adults. As crowding (“*a situation in which the identified need for emergency services outstrips available resources in the ED, hospital or both”*) is increasing in EDs worldwide [8, 9] prolonged LOS and unmet needs will expose older adults to increased risk of avoidable adverse events, such as loss of functional capacities and increased mortality [10–12]. Prolonged LOS for older adults in the ED is associated with increased risk of in-hospital adverse events [11]. Although this is well known, older adults have longer LOS in the ED than less vulnerable patient groups [12–15].

Frailty is a syndrome causing vulnerability when exposed to physiological and psychological stressors. Frailty in older adults is characterized by age-related decreased physiological capacity [16], but there is no common definition of the concept. Two main models, and related measurements, have been described to assess frailty: the phenotype model [16] and the cumulative deficit model [17]. The phenotype model is based on assessment of weight loss, exhaustion, weakness, slowness and reduced physical activity [16], whereas the cumulative deficit model is an index that is calculated based on symptoms (e.g., low mood), signs (e.g., tremor), abnormal laboratory values, disease states and disabilities/deficits [17].

According to consensus from an international expert group, all persons over the age of 70 should be screened for frailty, which is in line with recommendations from another group of experts in the field of frailty [18, 19]. Recently, clinical recommendations for geriatric emergency medicine have been developed by a group of clinical experts in Europe through a modified Delphi procedure [20]. The top two ranking activities proposed are comprehensive geriatric assessment and assessment of frailty [20].

There are several scales available for measuring frailty, and several have been tested in the ED [21]. The most prevalent tool, The Clinical Frailty Scale (CFS), is based on the cumulative deficit model [17] and is a clinical judgement–based frailty tool evaluating domains including comorbidity, function, and cognition [22]. Several scales have been used to estimate the prevalence of frailty and to predict adverse outcomes, of which mortality and admission to in-hospital care are most common [22–27]. Findings in a systematic review, that investigated factors associated with frailty, showed heterogeneity, including a great variety of factors and inconsistency in findings. Several risk factors were investigated, including age, gender, brain pathology and comorbidities. In particular, the impact of age and gender remains unclear and was suggested for further study [28].

Studies from ED settings across the globe have reported prevalence of frailty varying from 7 to 80% [25, 26, 29–33], but the results vary considerably due to the use of different measurement tools when screening for frailty, and varying age criteria for inclusion [23, 29]. The prevalence of frailty in older adults seeking emergency care in Sweden has rarely been reported; one study using a modified eight-item frailty phenotype method identified 72.3% of the participants aged 65 years or older as frail [34].

A study that investigated a screening tool in the ED to detect geriatric problems reported that 42.5% of eligible patients were screened. Reasons for excluding patients were life-threatening conditions and logistical reasons [35]. However, a more recent systematic review, based on four papers, reported that only 52% of eligible older patients in the ED were screened using any tool [21].

A more standardized assessment of frailty is needed, with appropriate tools that are simple, valid, not time-consuming, easy to use and acceptable to older adults and clinical staff [20, 29, 36]. In line with these recommendations, the FRail Elderly Support researcH group (FRESH) instrument was developed based on the phenotype model, with the aim to achieve an easy-to-use and valid instrument for screening in EDs. It consists of four questions to be answered by the older adult, as opposed to other scales that are based on assessment by the clinical staff [34].

### Rationale

As screening methods for frailty, tools, settings and included participants vary considerably, it is unknown how many older adults visiting EDs are at risk of being unattended based on their frailty condition. Early identification of frailty in older adults is valuable to improve awareness, establish priorities for care and identify older adults who would benefit from being screened. Thus, this study aims to describe the prevalence of, and factors associated with frailty in older adults seeking care at Swedish EDs.

## Method

### Design and setting

The study has a prospective cross-sectional design. Data were collected for one month, at three hospital-based EDs in Sweden, including one regional hospital (highly specialized care), one county hospital (most specialist clinics represented) and one rural hospital (only some specialist clinics represented). The EDs were chosen based on varying level and size of setting to achieve good representation of national ED care. The regional hospital had an annual patient flow of about 55,000, the county hospital 50,000 and the rural hospital 20,000 patients. Data at the rural hospital were collected in September 2019 and at the regional and the county hospital in November 2019.

The usual procedure on arrival to the ED is that the patient’s complaints are presented, and the patient’s level of urgency is assessed, referred to as triage. A registered nurse (RN) performs triage to determine how long it is medically safe for a patient to wait for an initial assessment by a physician, regardless of age. Thereafter, a physician on call in the ED assesses the patient and decides on diagnostic procedures and treatment.

### Research ethics

Participation was voluntary, and all data were handled confidentially. Personal data were de-identified and coded. The study was approved by the Swedish Ethical Review Authority (Dnr 2019/03650).

### Procedure

After ethics clearance, permission was granted by the managers of the EDs, and the researchers informed RNs at the EDs about the study at unit meetings. All RNs in the participating EDs were responsible for the inclusion, according to the inclusion criteria; patients aged 70 or older. Written and oral information about the study was provided to patients by RNs who did the inclusion when performing triage of patients on arrival to the EDs. Patients who agreed to participate signed a written consent form. The yes and no answers in the FRESH questionnaire were registered manually on a separate form, by the patients themselves or assisted by the attending RN, and the forms were not included in the patient records. All forms were collected every 24 h and were handed over to the researchers or the research assistant. During the data collection period, one RN/research assistant in the regional hospital–based ED and one RN/research assistant in the county hospital–based ED supported staff during the daytime with informed consent and inclusion of patients. An initial pilot test was undertaken in 2017, focusing on the feasibility of using the FRESH instrument, which guided the design of this study. As there were no frail patients identified in the age group 65–70 years in the initial pilot study, it was decided to change the inclusion criteria to patients 70 years and older.

### Sample

**Fig. 1:**
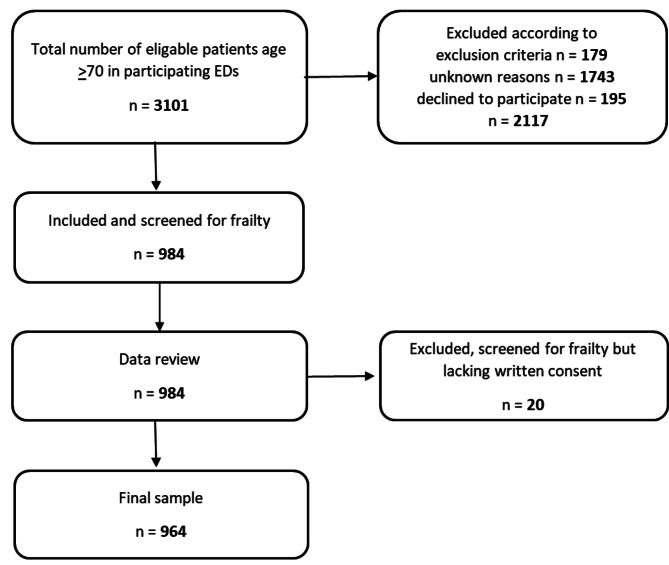
Flow chart of patient inclusion and exclusion at the ED

The sample consisted of older patients 70 years or older presenting at the EDs (Fig. 1). Exclusion criteria were time-sensitive medical conditions (e.g., myocardial infarction, stroke, severe trauma), unconsciousness, fast track patients (e.g., hip fractures, myocardial infarction). Further exclusion was based on cognitive impairment or other difficulties, such as language barriers, that did not allow patients to provide informed consent or made it difficult for them to respond to the assessment questions in the screening instrument upon arrival.

### Data collection

Data were collected using the FRESH instrument, which consists of four questions and response alternatives [34]. The FRESH instrument has been tested in a Swedish ED setting showing high specificity (80%) and sensitivity (81%), and a good discriminatory ability and usability (AUC = 0,862, 95% CI; 0,798-0,926.) [34].

1. “Do you get tired when taking a short (15–20 min) walk outside?”
2. “Have you suffered any general fatigue or tiredness over the last 3 months?”
3. “Have you fallen these last 3 months?”
4. “Do you need assistance to do your shopping?” (either getting to the store, or in choosing, paying for, or bringing home groceries?)

According to FRESH, patients were assessed as frail if they agreed with the assertions in two or more questions.

Data on patients’ characteristics (age, gender, living situation, triage acuity level, mode of arrival, weekday of arrival, main complaints,) and clinical outcomes (discharged or hospitalized, unplanned ED readmission within 72 h, LOS in the ED, time to treatment (TTT), that is, to be assessed by a physician, and mortality within 10 days) were collected from the electronic health records (EHR) and from the ED tracking systems via internal databases BILD (Business Intelligence Dalarna Region) and SAS visual analytics (Region Uppsala). The ED tracking system is a part of the EHR and visualizes all registered patients in the ED in real time. It contains information such as location, time of arrival and triage acuity level.

### Data analysis

Patient demographics and clinical outcomes were descriptively analysed as absolute and relative frequencies separately for frail and non-frail patients. Patients´ ages are presented as median values, percentiles, and range. Data on LOS and TTT were descriptively analysed using median values, percentiles, and range, since they were not normally distributed. SPSS version 27 for Windows was used to conduct descriptive analyses of patient demographics and clinical outcomes. Multivariable logistic regression was used to calculate odds ratios (OR), 95% Confidence Intervals (CI), and *p*-values for the association between frailty and the two clinical outcomes, admitted to inpatient care and new ED visit within 72 h; adjusted for age and gender. *P*-values < 0.05 were considered significant. Multivariable logistic regression was also used to model the association between patient demographic characteristics (age, gender, residence, and mode of arrival) and frailty. However, due to the high prevalence of frailty, estimates were presented as the marginal risk ratio (mRR) to facilitate interpretation [37]. The calculations were done using the statistical software R (emmeans 1.7.0 in R version 4.0.3).

Cox regression was used to calculate hazard ratio (HR), 95% CI, and *p*-values for the association between frailty and the two outcomes’ LOS and TTT, adjusted for age and gender. The calculations were done using the statistical software R (survival 3.4 − 0.1 in R version 4.0.3). Clinical experience and previous research [3, 6, 27] guided the selection and adjustment of variables.

## Results

Of the final sample of 964 patients, 553 (**57.3%)** were assessed as frail. Characteristics for the frail and non-frail group are depicted in Table 1.

**Table 1:** Description of characteristics for patients (n = 964) screened as frail or non-frail in the ED.

	Frail patients	Non-frail patients	Total
**Number included patients n (%)**	553 (57.3)	411 (42.7)	964
**Patients’ age median, years (1st and 3rd percentile)**	82 (76, 87)	77 (73, 81)	79 (75, 85)
Range	70–102	70–96	70–102
n (%)			
70–79	215 (38.9)	273 (66.4)	488 (50.6)
80+	338 (61.1)	138 (33.6)	476 (49.4)
**Gender, n (%)**			
Female	319 (57.7)	200 (48.7)	519 (53.8)
Male	234 (42.3)	211 (51.3)	445 (46.2)
**Residence, n (%)**			
Independent living	521 (94.2)	409 (99.5)	930 (96.5)
Residential care facility	32 (5.8)	2 (0.5)	34 (3.5)
**Triage acuity level n (%)**			
1 (Red) Immediate attendance	8 (1.4)	2 (0.5)	10 (1.0)
2 (Orange) Urgent	84 (15.2)	53 (12.9)	137 (14.2)
3 (Yellow) Semi-urgent	331 (59.9)	229 (55.7)	560 (58.1)
4 (Green) Non-urgent	68 (12.3)	66 (16.1)	134 (14.0)
5 (Blue) Not in need for triage	3 (0.5)	0 (0)	3 (0.3)
No triage level reported	59 (10.7)	61 (14.8)	120 (12.4)
**Arrival with EMS(**Emergency Medical Services, i.e., ambulance or helicopter staffed by paramedics) **transport, n (%)**			
Yes	198 (35,8)	97 (23.6)	295 (30.6)
No	355 (64.2)	314 (76.4)	669 (69.4)
**Time of arrival, n (%)**			
Day (7 a.m.–3:59 p.m.)	405 (73.2)	305 (74.2)	710 (73.7)
Evening (4p.m.–8:59 p.m.)	77 (13.9)	68 (16.5)	145 (15.0)
Night (9 p.m.–6:59 a.m.)	71 (12.8)	38 (9.2)	109 (11.3)
**Six most common chief complaints, n (%)**			
Abdominal pain	46 (8.3)	60 (14.6)	106 (11.0)
Chest pain	46 (8.3)	57 (13.9)	103 (10.7)
Dyspnoea	81 (14.6)	18 (4.4)	99 (10.3)
Injuries to extremities	60 (10.8)	39 (9.5)	99 (10.3)
Cardiac arrhythmia	33 (6.0)	30 (7.3)	63 (6.5)
Vertigo	27 (4.9)	25 (6.1)	52 (5.4)
Others	260 (47.0)	182 (44.3)	442 (45.9)

### Characteristics and significant risk factors for frailty

In this study, all modelled characteristics were significantly associated with frailty. Living in a *residential care facility* had the strongest association with a marginal risk ratio (mRR of 1.61 (95% CI: 1.41–1.84), *p* < 0.001), followed by *age > 80 years* (mRR = 1.52 (95% CI: 1.36–1.71), *p* < 0.001), *arriving to the ED with EMS* (mRR = 1.18 (95% CI: 1.05–1.32), *p* = 0.004) *and being a woman* (mRR = 1.13 (95% CI: 1.01–1.27), *p* = 0.029).

### The distribution of clinical outcomes between patients in the frail group and non-frail group

Clinical outcomes for the frail and non-frail group (n = 964) are depicted in Table 2.

**Table 2:** Description of clinical outcomes for frail or non-frail (n = 964) patients in the ED.

	Frail patients	Non-frail patients	Total
**Number included patients n (%)**	553 (57.3)	411(42.7)	964
**Admitted to in-hospital care, n (%)**			
Yes	268 (48.5)	124 (30.2)	392 (40.7)
No	285 (51.5)	287 (69.8)	572 (59.3)
**New ED visit within 72 h, n (%)**			
Yes	18 (3.3)	28 (6.8)	46 (4.8)
No	535 (96.7)	383 (93.2)	918 (95.2)
**Length of stay median, minutes (1st and 3rd percentile)**	321 (212, 526)	308 (194, 442)	319 (206, 499)
Range	35–1731	2–1684	2–1731
**Time to treatment median, minutes (1st and 3rd percentile)**	72 (31, 147)	70 (33, 141)	71 (32, 147)
Range	0–796	0–888	0–888
**Mortality within 10 days from ED visit, n (%)**			
Yes	5 (0.9)	0 (0)	5 (0.5)
No	548 (99.1)	411 (100)	959 (99.5)

### Clinical outcomes and significant risk factors for frailty

Frailty was significantly associated with admittance to in-hospital care (OR = 2.02 (95% CI: 1.53–2.68, *p* = < 0.001). There was no association between frail non-hospitalized patients and a new ED visit within 72 h (OR = 0.57 (95% CI: 0.28–1.12, *p* = 0.11).

The median LOS in the frail group was 321 min, and in the non-frail group 308 min, The median TTT was 73 min in the frail group and 70 min in the non-frail group. Resulting in no association between frailty (n = 553) and LOS (HR = 0.88 (95% CI: 0.77–1.0, *p* = 0.05) or with TTT (n = 551) (HR = 1.02 (95% CI: 0.89–1.16, *p* = 0.82).

## Discussion

Our study contributes with new knowledge concerning prevalence of frailty and demographic characteristics associated with frailty in older adults seeking ED care. Our results showed that the prevalence of frailty in patients aged ≥ 70 years was 57.3%. A scoping review reported that several studies from various countries have estimated the prevalence of frailty in the ED, using a variety of tools and inclusion criteria, showing considerable variation, from 7 to 80%, with a median prevalence of 47%, in populations of older adults [29].

The prevalence of frailty in our study is based on assessment of patients using the FRESH instrument. Most studies evaluating screening and management of frailty in the ED considered age ≥ 65 years as older adults, and the screening was usually based on clinical judgement by clinical staff [38]. It has been reported that clinical staff tend to assess older adults as frail to a higher extent than the older adults themselves [39, 40].

To our knowledge, only one previous Swedish study has estimated the prevalence of frailty in an ED setting using the FRESH instrument [34]. In that study the prevalence was estimated at 73%, which was based on 161 older adults aged 65 years or older with at least one chronic disease and dependency in at least one activity of daily living. The higher prevalence of frailty in that study compared to ours might be explained by the smaller sample of older adults and the more restricted inclusion criteria. Further, the frailty assessment was conducted after returning home from the ED as opposed to our assessment on arrival to the ED [34].

As the knowledge about the accuracy of tools for identifying frailty is limited, results from studies cannot easily be compared and generalized due to the heterogeneity of assessment tools, inclusion criteria and settings [37]. The European Task Force on Geriatric Emergency Medicine has recently recommended to incorporate mobility and/or frailty assessment in ED triage systems [20]. The FRESH instrument has been validated and found easy to use in Swedish ED settings; further, the questions are commonly asked during triage and easy to answer. Therefore, it should be quite easy to include these questions in any triage system.

In our study, only 32% of eligible older adults were screened for frailty, other studies have reported about 52% of eligible older adults being screened [21, 38]. However, in our study, the exclusion criteria, for example, dementia, might be one explanation to why not all eligible patients were screened. There were several patients who were included and triaged with the most and second most urgent acuity level, i.e., unstable patients. There was also an association between the frail group and arriving to the ED with EMS. This indicates that older persons seeking care in the ED are in need for such care. If there had been a wider inclusion of older adults for screening, and the FRESH instrument had been supplemented with screening for cognitive impairment, the prevalence of frailty might have been higher in our study. Frailty is significantly associated with dementia according to a study in a geriatric department including a frailty status with a short emergency geriatric assessment tool (SEGA) [41]. In our study, presenting complaints differed between the frail and non-frail group. Dyspnoea was more common in the frail group, which has also been reported from another study, that also reported general weakness to be associated with frailty at ED arrival [42].

In our study, patient characteristics significantly associated with frailty were living in a residential care facility, which had the strongest association with frailty, followed by age > 80 years, arriving to the ED with EMS and being a woman. Clinical outcomes with significant association to frailty were admittance to in-hospital care.

The characteristic with the strongest association with frailty in our study was living in a residential care facility, which has also been reported in previous studies [41, 43]. Association between frailty, older age and being a woman has also been reported in a previous study [44]. Further, clinical outcomes associated with frailty in our study were admittance to in-hospital care, but there was no association between frail non-hospitalized patients and a new ED visit within 72 h. A Swedish study [45] based on registry data from two regions, evaluated the association between patient characteristics and ED visits and revisits within 30 days among patients aged 65 years or older. Nearly one patient out of five in that study had an ED revisit within 30 days and between 1% and 3% revisited the same day as having been discharged from the ED [45]. However, it is not possible to make comparisons between their results and our study because we used different time frames for revisits. Our finding that age ≥ 80 years and being a woman was associated with frailty confirms findings from a previous study using the frail safe questionnaire [46].

Arriving with EMS to the ED was also associated with frailty in our study, which coheres with a review reporting that 76–77% of frail older adults arrived with EMS to the ED [43]. A Swedish study aimed to describe characteristics of older adults aged 70 or older compared with adults aged 18–69, requiring EMS. Of the included EMS transfers, 59.9% were older adults aged 70 or older and were more likely to be transported to hospital. In addition, older adults in this study had a significantly lower probability to receive the highest triage priority compared to adults aged 18–69 [47]. Based on our results and these two studies [43, 47], it might be justified that screening for frailty should be done prehospital, before arrival to ED, and that triage systems should consider older age and frailty.

Frailty was not associated with LOS in the ED and TTT; however, frailty might have been associated with LOS if the sample of included patients had been larger. There are other studies that have reported on frailty associated with increased LOS during in-hospital care [24, 46, 48], which should be investigated further. If frailty is associated with increased LOS during in-hospital care, it is even more important to identify frail older adults as soon as possible to avoid unnecessary risks and harm. Further, our results show a significant association between frailty and admittance to in-hospital care, which is in line with several studies that have reported that frailty is associated with adverse outcomes, most commonly mortality and admittance to in-hospital care [22–24].

### Strengths and limitations

This study contributes with knowledge about the prevalence of frailty in older adults in the ED, and highlights some of the methodological challenges in studying this phenomenon in the busy ED setting. Of eligible patients, only 32% were included and screened for frailty, resulting in a considerable dropout, which compromised the inclusion of patients. Patients were included mostly during the daytime; the reason might be that two of the EDs had an RN/research assistant available to support data collection during the daytime, but not during evening and night shifts. It may also be an indication that older adults mostly seek care during daytime. Other reasons might be, as shown in previous studies, shortage of staff in the evenings and nights, and the fast-paced nature of ED care that may obstruct data collection. Further, the documentation of the assessment was done manually and not included in the EHR, which may have been perceived as extra work for the RNs in the triage. Further, patients with cognitive impairment, patients with difficulties to understand and answer the questions and patients with time-sensitive-conditions were excluded. If these patients had been included, the prevalence of frailty might have been higher. Even though our study confirms results from previous research using different tools, the FRESH instrument should be supplemented with screening tools for cognitive impairment, as suggested by expert clinical recommendations for geriatric emergency medicine [20].

As the study was conducted at three EDs in two regions, representing various levels of ED care and urban and more rural populations, it can be assumed that the data provide a good representation of ED care in Sweden. However, generalizability to other countries is dependent on demography, and should be done with caution.

## Conclusion

This study shows a high prevalence of frailty in older adults seeking ED care. Factors associated with frailty were living in a residential care facility, age ≥ 80 years, being a woman and arriving with EMS to the ED and being admitted to in-hospital care. Frailty screening should be incorporated in the triage system for an early identification of frail patients who need tailored interventions and to avoid unnecessary risk and harm. More studies using the FRESH instrument are needed to further confirm our findings and to develop the methods for screening for frailty in the ED.

## Data Availability

The data that support the findings of this study are not openly available due to reasons of sensitivity and are available from the corresponding author upon reasonable request.

## Abbreviations

FRESH: FRail Elderly Support research group
EMS: emergency medical service
ED: Emergency Department
LOS: Length of stay
TTT: Time To Treatment
CFS: Clinical Frailty Scale
RN: Registered Nurse
HER: Electronic health record
